# Bone Marrow Aplasia Under Tyrosine Kinase Inhibitors in Chronic Myeloid Leukemia: A Rare and Unpredictable Complication

**DOI:** 10.7759/cureus.117060

**Published:** 2026-09-28

**Authors:** Morad Annasser, Omar Halloumi, Rida Fatima ezzahra, Salma Fares

**Affiliations:** 1 Hematology, Centre Hospitalier Universitaire Mohammed VI, Agadir, MAR; 2 Clinical Hematology and Cellular Therapy, Centre Hospitalier Universitaire Mohammed VI, Agadir, MAR; 3 Hematology, Hôpital Universitaire de Bruxelles (HUB) Institut Jules Bordet, Bruxelles, BEL; 4 Hematology, Ibn Zohr University, Medical School of Agadir, Agadir, MAR; 5 Hematology, Faculté de Médecine et de Pharmacie Agadir, Agadir, MAR

**Keywords:** bone marrow aplasia, chronic myeloid leukemia, gelatinous marrow degeneration, imatinib, nilotinib, pancytopenia, tyrosine kinase inhibitors

## Abstract

Tyrosine kinase inhibitor (TKI)-induced bone marrow aplasia is a rare but potentially severe complication of chronic myeloid leukemia (CML). We report two cases occurring under imatinib and nilotinib in patients with chronic-phase CML. The first case, occurring six months after imatinib initiation, was characterized by severe bone marrow aplasia associated with gelatinous marrow transformation, a rare histological finding in TKI-treated CML. The second case, occurring two months after switching to nilotinib, evolved toward non-severe bone marrow aplasia, with complete hematologic recovery after treatment discontinuation. These observations illustrate the heterogeneity and unpredictability of this toxicity, which may occur across different TKI generations and levels of molecular response. Immediate TKI discontinuation remains essential, and any persistent pancytopenia should prompt early bone marrow evaluation to ensure a timely diagnosis and appropriate management.

## Introduction

Bone marrow aplasia secondary to tyrosine kinase inhibitors (TKIs) in chronic myeloid leukemia (CML) is an exceptional complication, estimated to affect fewer than 1% of imatinib-treated patients [1], potentially severe and likely underrecognized. Chronic myeloid leukemia is a clonal myeloid neoplasm resulting from the t(9;22)(q34;q11) translocation, generating the BCR::ABL1 fusion gene responsible for a constitutively activated TKI and the uncontrolled proliferation of myeloid precursors [2]. The advent of TKIs has radically improved the outlook of the disease: imatinib, the first approved TKI, achieves complete hematologic response rates exceeding 95% in chronic phase [3], while nilotinib, a second-generation TKI approximately 30-fold more potent than imatinib [4], is active against most imatinib-resistance mutations except T315I [5].

Unlike the relatively common, usually reversible cytopenias observed during TKI therapy, true bone marrow aplasia is characterized by profound marrow hypocellularity and may persist despite treatment interruption. This complication nevertheless remains rare and incompletely characterized: its pathophysiological mechanism is not well elucidated, its management is not standardized, and comparative data across TKI generations are largely lacking.

In this context, we report two cases of bone marrow aplasia occurring under imatinib and nilotinib, respectively. These observations are notable for the identification of gelatinous marrow transformation, a rare histological alteration characterized by replacement of the normal marrow stroma by gelatinous extracellular material, previously reported in CML patients receiving TKI therapy but rarely described in this setting [6-8]. We analyze their clinical features, course, and management in light of the available literature.

## Case presentation

Case #1

Bone Marrow Aplasia Under Imatinib

A 31-year-old man with no notable medical history was diagnosed with chronic-phase CML based on hyperleukocytosis associated with splenomegaly, discovered during work-up for febrile abdominal pain. Initial work-up revealed marked hyperleukocytosis at 461,000/mm³ (normal range, 4,000-10,000/mm³), 50% myelemia, severe anemia with hemoglobin of 7.6 g/dL (normal range, 13-17 g/dL), and thrombocytosis at 481,000/mm³ (normal range, 150,000-400,000/mm³). Bone marrow aspirate and karyotype, showing the t(9;22) translocation, confirmed the diagnosis of chronic-phase CML. The Sokal score (0.73) and Hasford score (584.51) classified the patient as low risk, with no indication for upfront second-generation TKI therapy.

Imatinib 400 mg/day was initiated. At three months, molecular assessment showed a BCR::ABL1 transcript level of 24% (IS), indicating failure to achieve the early molecular response milestone (≤10%), despite an achieved hematologic response [9]. After six months of therapy, the patient developed severe pancytopenia, including grade 4 neutropenia and thrombocytopenia per the National Cancer Institute Common Terminology Criteria for Adverse Events (NCI-CTCAE) version 5.0 [10], with hemoglobin of 6.6 g/dL, a white blood cell count of 630/mm³ with an absolute neutrophil count (ANC) of 90/mm³, lymphocytes of 520/mm³, platelets of 6,000/mm³, and a reticulocyte count of 9,400/mm³. No myelemia or blasts were observed on the peripheral blood smear. The laboratory findings at the time of aplasia, together with the corresponding reference ranges, are summarized in Table 1.

**Table 1:** Laboratory findings of case #1 at the time of bone marrow aplasia, with corresponding reference ranges Values were obtained at the time of bone marrow aplasia, six months after initiation of imatinib. ᵃLast molecular assessment available before the onset of aplasia. The reference ranges provided in this table are based on the standard reference intervals used by our laboratory. ANA: Antinuclear antibodies, HBV: Hepatitis B virus, HCV: Hepatitis C virus, IS: International scale, RF: Rheumatoid factor

Parameter		Reference range (WHO)	Result
Peripheral blood counts	Hemoglobin	13.0-17.0 g/dL	6.6 g/dL
	WBC count	4,000-10,000/mm³	630/mm³
	Absolute neutrophil count	1,500-7,000/mm³	90/mm³
	Lymphocyte count	1,000-4,000/mm³	520/mm³
	Platelet count	150,000-400,000/mm³	6,000/mm³
	Reticulocyte count	25,000-75,000/mm³	9,400/mm³
	Myelemia	0%	0%
	Circulating blasts	0%	0%
	Bone marrow		
	Marrow cellularity	Age-adjusted (≈100 − age, %)	15-20%
	Blast excess on trephine biopsy	Absent	Absent
Cytogenetics and molecular biology	Karyotype	46,XY	Not performed
	BCR::ABL1 transcript (international scale (IS))	Undetectable	24% (month 3)ᵃ
Etiologic work-up	HIV, hepatitis B virus (HBV), hepatitis C virus (HCV) serologies	Negative	Negative
	Antinuclear antibodies (ANA), rheumatoid factor (RF)	Negative	Negative
	Vitamin B9, B12	Within normal limits	Normal

The bone marrow aspirate was markedly paucicellular and initially raised concern for an increased blast proportion; however, its interpretation was limited by the very low number of evaluable cells. Bone marrow biopsy, however, revealed a markedly hypocellular marrow (cellularity 15-20%) without tumor infiltration or blast excess, confirming a diagnosis of very severe aplastic anemia according to Camitta's criteria [11,12], with marrow cellularity below 25% and all three peripheral blood criteria met (ANC <500/mm³, platelets <20,000/mm³, and reticulocytes <20,000/mm³), including an ANC <200/mm³, which defines the very severe category. Gelatinous marrow transformation was also present, a rare histological finding previously reported in CML patients receiving TKI therapy (Figure 1).

**Figure 1:**
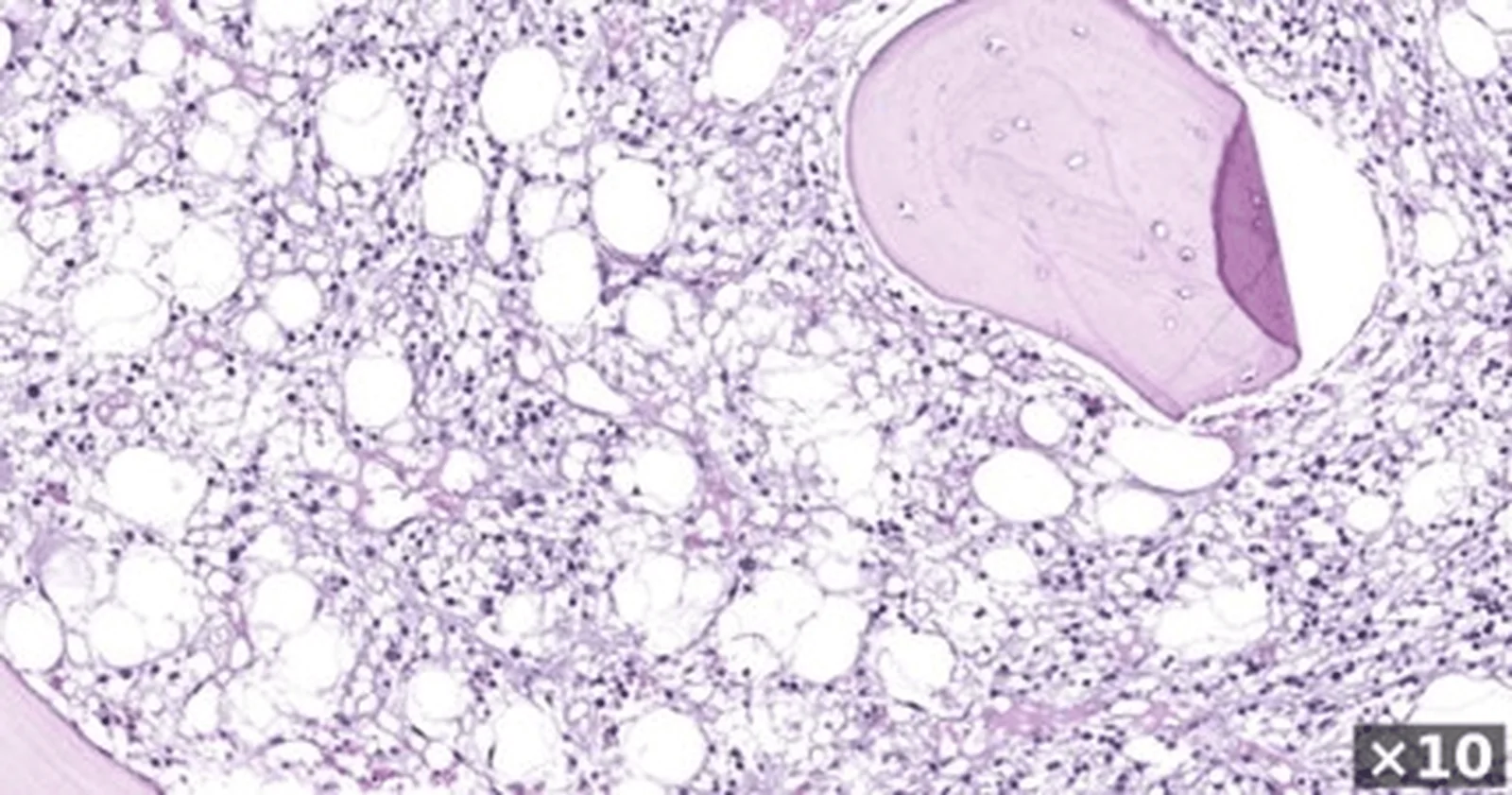
Bone marrow biopsy showing a marrow space filled with an amorphous gelatinous substance, within which rare isolated hematopoietic elements persist (H&E).

The etiologic work-up, performed to exclude differential diagnoses, included viral serologies (HIV, hepatitis B and C), which returned negative; a negative autoimmune panel (ANA, RF); and vitamin assays (B9, B12) without abnormality. This work-up did not identify an infectious, autoimmune, or nutritional alternative cause. Bone marrow karyotyping could not be performed, representing a limitation of our observation.

From a pathophysiological standpoint, the persistence of a detectable BCR::ABL1 transcript on the last molecular assessment available before the onset of aplasia argues against documented eradication of the leukemic clone and may support a direct bone marrow toxicity of imatinib via inhibition of c-KIT and PDGFR. Gelatinous marrow transformation, defined by replacement of the hematopoietic matrix with an amorphous mucoid substance, reflects profound remodeling of the hematopoietic niche that may have contributed to the absence of recovery at five months.

The clinical course was complicated by febrile neutropenia with neutropenic colitis confirmed by abdominal computed tomography. Blood cultures and stool cultures remained sterile. The patient received imipenem and metronidazole for eight days, with resolution of fever by day two of antibiotic therapy.

Supportive care included transfusion of 12 units of packed red blood cells (PRBC) and 32 units of platelets. No granulocyte colony-stimulating factor (G-CSF) was administered, given the lack of demonstrated benefit of growth factors in TKI-induced bone marrow aplasia in which global hypocellularity reflects progenitor-cell injury rather than a maturation defect, together with the theoretical risk of stimulating the residual leukemic clone, which remained detectable in this patient. At the fifth-month follow-up after imatinib discontinuation, no significant marrow recovery had been observed. Regarding the therapeutic plan, management relied on imatinib discontinuation and supportive care, with close hematologic monitoring. Given the absence of recovery at five months, cyclosporine-based immunosuppression and allogeneic hematopoietic stem cell transplantation (HSCT) were discussed and retained as options should non-recovery persist.

Case #2

Bone Marrow Aplasia Under Nilotinib

A 62-year-old woman with no notable medical history had been followed for chronic-phase CML for eight years. After a period of irregular follow-up, she was restarted on imatinib 400 mg/day for approximately eighteen months. Molecular assessment showed a BCR::ABL1 transcript level of 8.86% (IS) after approximately 18 months of imatinib therapy, resumed following a period of irregular follow-up. This value exceeds the threshold generally associated with treatment failure beyond 12 months according to the 2025 European LeukemiaNet (ELN) recommendations [9], although the preceding irregular follow-up limits definitive classification of the response trajectory. The Sokal score (1.21, high risk) and Hasford score (1,010, intermediate risk) warranted intensified hematologic monitoring during the switch to nilotinib.

Two months after nilotinib initiation, the patient developed severe cytopenias, with a hemoglobin of 7.2 g/dL, a white blood cell count of 4,500/mm³ with an ANC of 576/mm³, and a platelet count of 35,000/mm³. The neutropenia and thrombocytopenia were grade 3 according to NCI-CTCAE v5.0 [10]. The reticulocyte count was 26,000/mm³, and the peripheral blood smear was unremarkable. The laboratory findings at the time of aplasia, together with the corresponding reference ranges, are summarized in Table 2.

**Table 2:** Laboratory findings of case #2 at the time of bone marrow aplasia, with corresponding reference ranges ANA: Antinuclear antibodies, HBV: Hepatitis B virus, HCV: Hepatitis C virus, IS: International scale, RF: Rheumatoid factor Values were obtained at the time of bone marrow aplasia, two months after the switch to nilotinib. ᵃLast molecular assessment available before the switch to nilotinib. The reference ranges provided in this table are based on the standard reference intervals used by our laboratory.

Parameter		Reference range	Result
Peripheral blood counts	Hemoglobin	12.0-15.0 g/dL	7.2 g/dL
	White blood cell count	4,000-10,000/mm³	4,500/mm³
	Absolute neutrophil count	1,500-7,000/mm³	576/mm³
	Lymphocyte count	1,000-4,000/mm³	3,784/mm³
	Platelet count	150,000-400,000/mm³	35,000/mm³
	Reticulocyte count	25,000-75,000/mm³	26,000/mm³
	Myelemia	0%	0%
	Circulating blasts	0%	0%
Bone marrow	Marrow cellularity	Age-adjusted (≈100 − age, %)	≈10% (≈90% adipose replacement)
	Blast excess on trephine biopsy	Absent	Absent
Cytogenetics and molecular biology	Karyotype	46,XX	Not performed
	BCR::ABL1 transcript (IS)	Undetectable	8.86% (before switch)ᵃ
Etiologic work-up	HIV, HBV, HCV serologies	Negative	Negative
	ANA, RF	Negative	Negative
	Vitamin B9, B12	Within normal limits	Normal

The bone marrow aspirate was poor and poorly contributory. Bone marrow biopsy confirmed the diagnosis, showing a markedly hypoplastic marrow largely replaced by adipose tissue, with residual persistence of all three hematopoietic lineages, without morphological atypia or tumor infiltration, consistent with non-severe bone marrow aplasia [11,12], as the peripheral blood criteria for severe aplastic anemia were not met (Figure 2). The etiologic work-up for the bone marrow aplasia was unremarkable; karyotyping could not be performed. Management relied on supportive care with the transfusion of two units of PRBCs. No G-CSF was administered, as the neutropenia (ANC 576/mm³) did not warrant its use. At the seventh-month follow-up after nilotinib discontinuation, a progressive improvement in blood counts was observed, resulting in complete normalization. The patient was subsequently started on dasatinib 50 mg twice daily, under close hematologic monitoring.

**Figure 2:**
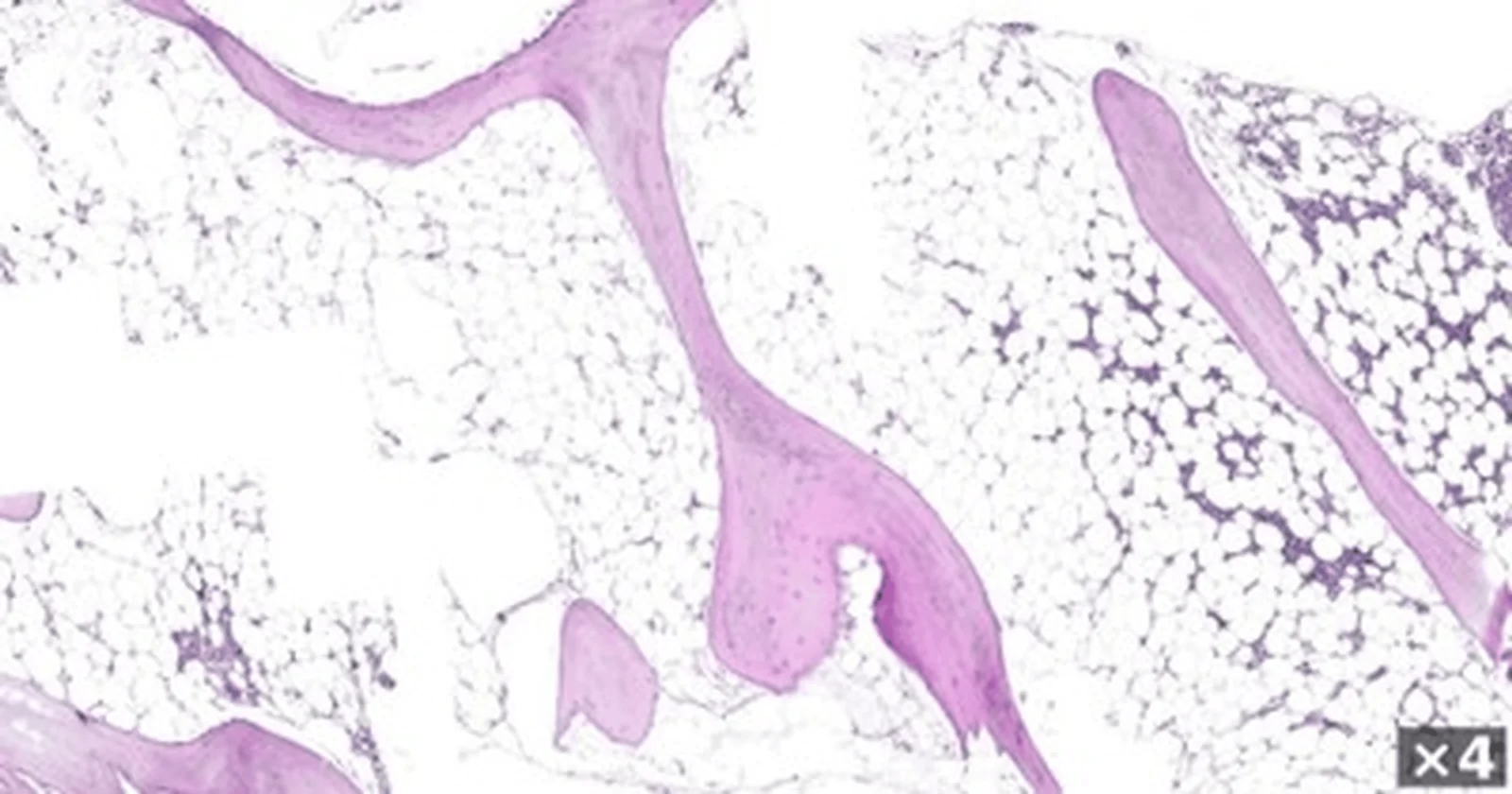
Bone marrow biopsy showing four marrow spaces with massive adipose replacement, estimated at 90%, with persistence of rare residual hematopoietic cells (H&E).

## Discussion

Chronic myeloid leukemia is a clonal hemopathy resulting from the t(9;22) translocation, giving rise to the constitutively activated BCR::ABL1 tyrosine kinase. By targeting this kinase domain, imatinib has profoundly altered the natural history of the disease, with a 10-year overall survival of 83.3% in the International Randomized Study of Interferon and STI571 (IRIS) trial [13]. Beyond their anti-BCR::ABL1 activity, imatinib and nilotinib also inhibit the c-KIT and PDGFR kinases, which are involved in the regulation of normal hematopoietic progenitors, constituting a potential substrate for their marrow toxicity [14].

Transient cytopenias are among the most frequent hematologic adverse events during TKI therapy and are generally reversible with treatment interruption or dose adjustment. True bone marrow aplasia represents a distinct and substantially rarer complication, defined by global marrow hypocellularity without tumor infiltration, resistant to simple dose reduction. Several mechanisms have been proposed: direct inhibition of stem cells via c-KIT and PDGFR, an autoimmune response through molecular mimicry, and dose-dependent toxicity related to metabolic polymorphisms [14,15]. The exact mechanism remains unelucidated; the autoimmune hypothesis, suggested on the basis of additional cytogenetic abnormalities in Philadelphia-negative cells, could explain certain forms resistant to TKI discontinuation alone [15].

Bone marrow aplasia under TKIs is a rare complication, estimated at fewer than 1% of imatinib-treated patients [1]. Since the first published descriptions from 2005 onward [16,17], the number of reported cases has remained limited, giving each new case additional scientific and clinical value. The two cases reported here, occurring under imatinib and nilotinib and showing distinct patterns of marrow aplasia and recovery, add to this limited body of evidence, as summarized alongside previously published cases in Tables 3-4.

Time to onset is a key diagnostic feature. Under imatinib, it ranges from a few weeks to more than a year, with a median of 3.3 months [1]. Our first case, occurring at six months, falls within this range [16,18,19]. The late-onset case reported by Khan et al. demonstrates that the risk window is not confined to the early months of treatment [20]. With nilotinib, the toxicity window is earlier and narrower, typically occurring within the first two months [21-23], as our second case illustrates. Severe pancytopenia is the dominant clinical presentation in nearly all published cases, as shown in Table 3.

**Table 3:** Published cases of bone marrow aplasia associated with imatinib therapy Allo-HSCT: Allogeneic hematopoietic stem cell transplantation, CML: Chronic myeloid leukemia, EPO: erythropoietin, G-CSF: Granulocyte colony-stimulating factor, PRCA: Pure red cell aplasia, MMR: Major molecular response,TKI: Tyrosine kinase inhibitor

Parameter	Lokeshwar et al., 2005 [16]	Srinivas et al., 2007 [17]	Cervetti et al., 2008 [15]	Khan et al., 2008 [20]	Poudyal et al., 2016 [21]	Ramdial et al., 2019 [19]	Madabhavi et al., 2023 [1]	Dogra et al., 2024 [18]	Erol et al., 2024 [14]	Case #1 (present study)
Time to onset	Six weeks	Three to six months	~Three months	19 months	Four months	Eight months	Median 3.3 months (1.5-6 months)	Four months	~10 months	Six months
Clinical presentation	Pancytopenia + fever + hemorrhage	Severe pancytopenia	Progressive pancytopenia	Pancytopenia + fever	Isolated anemia (PRCA)	Severe pancytopenia	Severe pancytopenia	Severe pancytopenia	Severe pancytopenia	Severe pancytopenia
Marrow findings	Aplasia <5%	Aplasia 5% to 10%	Aplasia + cytogenetic abnormalities	Bone marrow aplasia	Pure red cell aplasia (PRCA)	Bone marrow aplasia	Severe hypocellularity	Severe hypocellularity	Bone marrow aplasia	Very severe aplasia + gelatinous transformation
BCR::ABL1 status	Not specified	Positive	Positive	0% Philadelphia chromosome + (complete cytogenetic response)	Major molecular response (MMR)	Positive	Positive (several cases)	Positive	Positive (49%)	Positive (24%)
Management	Discontinuation + antibiotics + G-CSF	Imatinib discontinuation	Discontinuation + erythropoietin + allo-HSCT	Discontinuation + antibiotics + anti-tuberculosis therapy	Discontinuation + steroids	Imatinib discontinuation	Discontinuation + supportive care	Discontinuation + supportive care	Discontinuation → TKI switch → allo-HSCT evaluated	Discontinuation + supportive care
Outcome	Death (mucormycosis)	One death (cerebral hemorrhage); four followed	Recovery (cytogenetic abnormalities)	Death (tuberculosis)	PRCA recurrence upon rechallenge	Recovery after discontinuation	Median recovery 10 months	Progressive recovery	Persistent cytopenias under 2nd/3rd-line TKI	No recovery at five months

Our first case is distinguished by the occurrence of neutropenic colitis, a visceral complication rarely reported in this setting, and by the presence of gelatinous marrow transformation on a bone marrow biopsy. Although gelatinous marrow transformation has previously been reported in CML patients receiving TKIs, including imatinib and dasatinib, it remains a rare and poorly characterized finding in this setting [6-8]. This lesion, defined by replacement of the hematopoietic matrix with an amorphous, glycosaminoglycan-rich mucoid substance, is classically associated with severe malnutrition or anorexia but also with certain drug toxicities. Its presence in our observation, in the absence of any nutritional deficiency, suggests a direct stromal injury to the hematopoietic niche, reflecting severe marrow cellular stress that may have aggravated and prolonged the aplasia.

From a mechanistic standpoint, additional cytogenetic abnormalities in Philadelphia-negative cells, suggestive of an autoimmune mechanism, have also been reported in the literature [15]. In both of our cases, bone marrow karyotyping was not repeated at the time of aplasia to assess for such abnormalities, a limitation that precluded exploration of this pathophysiological hypothesis. Finally, the clinical spectrum extends beyond global aplasia and includes focal forms such as isolated pure red cell aplasia (PRCA) [21], as illustrated in Table 4.

**Table 4:** Published cases of bone marrow aplasia associated with nilotinib therapy MMR: Major molecular response, PRCA: Pure red cell aplasia, TKI: Tyrosine kinase inhibitor

Parameter	Song et al., 2009 [22]	Prodduturi et al., 2012 [24]	Estephan et al., 2014 [23]	Poudyal et al., 2016 [21]	Case #2 (present study)
Time to onset	~Two months	Six months	2.5 months	~One to two months	Two months
Clinical presentation	Pancytopenia + gastrointestinal hemorrhage	Severe pancytopenia	Severe pancytopenia	Isolated anemia (PRCA)	Severe pancytopenia
Marrow findings	Aplasia <5%	Severe hypocellularity	Aplasia <5%	PRCA	Non-severe bone marrow aplasia
BCR::ABL1 status	Positive	Positive	Positive (low level)	MMR	Positive (8.86%)
Management	Nilotinib discontinuation	Nilotinib discontinuation	Nilotinib discontinuation	Discontinuation + immunosuppressants	Nilotinib discontinuation + supportive care
Outcome	Partial recovery	Recurrence upon reintroduction	Recovery after 5 months	Controlled with cyclosporine	Full recovery at 7 months

In both cases, BCR::ABL1 remained detectable on the last molecular assessment available before the onset of aplasia [1,17,18]. Together with previously reported cases occurring during major molecular or complete cytogenetic response [20,21,24], these observations illustrate that TKI-associated marrow aplasia may occur across different levels of molecular response. Additionally, higher imatinib doses (600-800 mg) appear to be associated with earlier onset of aplasia, suggesting a dose-dependent effect [17].

Immediate TKI discontinuation is the recommended first therapeutic step. Our first patient showed no marrow recovery at five months, below the median recovery time of 10 months reported in the literature [1], raising the question of therapeutic escalation. Cyclosporine-based immunosuppression [21] and allogeneic hematopoietic stem cell transplantation [14,15] represent potential therapeutic options in cases of failure to spontaneously recover, or of aplasia occurring with multiple TKIs. Our second patient achieved recovery at seven months under supportive care alone, an outcome comparable to cases described in the literature [23]. Reintroduction of the responsible TKI should be avoided given the high risk of recurrence or fatal outcome [16,24].

Our two cases illustrate that this complication may occur across different risk profiles and treatment lines in distinct clinical contexts. They highlight, in a North African setting where access to salvage therapies remains limited, the importance of early detection. Weekly hematologic monitoring during the first two months under nilotinib and monthly monitoring during the first six months under imatinib is recommended. Any severe, persistent pancytopenia mandates bone marrow biopsy before attributing the cytopenia to drug toxicity alone. Reintroduction of the same molecule should be avoided given the risk of recurrence [14,24]. In the absence of spontaneous recovery, cyclosporine-based immunosuppression [21] or allogeneic hematopoietic stem cell transplantation [14,15] should be considered according to the clinical context and available resources.

This report has several limitations. First, it is based on two observations only, which precludes any estimation of incidence or generalization of the findings. Second, bone marrow karyotyping could not be repeated at the time of aplasia in either patient, so that additional cytogenetic abnormalities in Philadelphia-negative cells could not be sought and the autoimmune hypothesis could not be explored. Third, TKI plasma level monitoring was not available in our setting, precluding assessment of a possible dose-dependent contribution to the observed toxicity. Fourth, follow-up remains short in the first case, in whom marrow recovery had not occurred at five months, so that the final hematologic outcome cannot yet be established. Finally, the rarity of this complication means that no standardized management recommendations currently exist; the therapeutic strategies discussed here derive from isolated case reports and small series rather than from prospective comparative data and should therefore be interpreted with caution.

## Conclusions

Bone marrow aplasia under TKIs, although rare, may occur across different levels of molecular response, mandating early diagnosis through close hematologic monitoring and systematic bone marrow biopsy for any persistent, severe pancytopenia. Immediate TKI discontinuation together with supportive care remains the cornerstone of management. In the absence of spontaneous recovery, cyclosporine-based immunosuppression or allogeneic hematopoietic stem cell transplantation should be considered.
